# The impact of guided meditations and mindfulness on blood pressure regulation: analyzing the physiological impact of mental health techniques on cardiac biomarkers

**DOI:** 10.1080/28324765.2025.2535718

**Published:** 2025-07-22

**Authors:** Gayathri Gururamalingam, Taruna Ramnath, Namita Ruhela, Rohit Jain

**Affiliations:** a Department of Medicine, Karnataka Institute of Medical Sciences, Hubballi, KA, IN; b Department of Medicine, Trinity School of Medicine, VC; c Department of Medicine, Aureus University School of Medicine, Oranjestad, AW; d Department of Internal Medicine, Penn State Health Milton S. Hershey Medical Center, Hershey, PA, USA

**Keywords:** Hypertension, mindfulness based interventions, mindfulness, guided meditation, mental health, heart rate variability

## Abstract

Hypertension is a significant preventable risk factor for cardiovascular disease and overall mortality globally, affecting 31.1% of the adult population in 2010. The prevalence of hypertension is rising due to aging and lifestyle factors such as poor diets and insufficient physical activity. Conventional therapy modalities often encompass physical or pharmacological measures; nevertheless, the mental practice of meditation has been employed for centuries to reduce cardiovascular risk. However, the scientific revitalization of this technique has previously prompted doubts over its legitimacy. This paper examines the effects of guided meditations and mindfulness techniques on blood pressure regulation and their potential to enhance cardiac biomarkers, including heart rate variability, cortisol levels, and inflammatory markers, through a review of existing literature and empirical studies. The findings suggest that mindfulness and guided meditation may serve as promising, non-pharmacological complements to traditional hypertension and cardiovascular disease treatments, though further research is needed to strengthen these conclusions.

## Introduction

Hypertension is a vascular condition in which blood pressure (BP) continuously exceeds a certain threshold. A systolic blood pressure (SBP) of 130–139 mmHg and a diastolic blood pressure (DBP) of 80–89 mmHg are considered stage 1 hypertension, per the American College of Cardiology (ACC)/American Heart Association’s (AHA) guidelines (Flack & Adekola, 2020). The prevalence of high blood pressure is rising across the globe as the population ages and more people are exposed to lifestyle risk factors such as poor diets (low potassium and high sodium intake) and a lack of exercise (Mills et al., 2020). The prevalence varies by geographical location and income level. The WHO reports that the region of the Americas has the lowest prevalence of hypertension (18%), while the African region has the highest (27%) (World Health Organization, 2023). In low- and middle-income countries, the number of adults with hypertension grew from 594 million in 1975 to 1.13 billion in 2015. High blood pressure can cause significant cardiovascular diseases such as, stroke, myocardial infarction, chronic renal disease, heart valve problems, coronary artery disease, cardiac arrest, and abdominal aortic aneurysm, potentially resulting in premature death and disability (World Health Organization, 2021). As a result, high blood pressure management and prevention strategies must be enhanced while treating hypertension.

Currently, hypertension treatment consists of a combination of medications and lifestyle adjustments (Li et al., 2021). Antihypertensive drugs are commonly used to reduce high blood pressure, but the incidence of uncontrolled BP remains high due to medical expenses, difficulties adhering to treatment, and adverse effects (World Health Organization, 2021). Lifestyle changes such as quitting smoking, eating a balanced diet, exercising regularly, practicing mindfulness meditation, and engaging in behavioral therapy have emerged as the primary non-pharmacological first-line treatment for hypertension (Mir et al., 2024). These adjustments can lower high blood pressure as effectively as a single antihypertensive medicine.

Implementing mindfulness-based meditation (MBM), interventions combine self-compassion modification as an emotional distress alleviation to improve mental health by relaxing emotional barriers and stress alleviation (Verma et al., 2021). Mir et al. demonstrated a link between MBM and hypertension, citing stress and depression as major factors to hypertension. They conducted a database search of randomized control trials (RCT), which found nine studies with 543 participants, with eight reporting MBM as effective in lowering systolic BP and six in reducing diastolic BP (Mir et al., 2024). However, previous research has yet to focus on the impact of mindfulness and guided meditation practice in cardiology, particularly their effects on cardiac indicators. In this study, we examine the influence of mindfulness and guided meditation on blood pressure and cardiac health. We focus on how mindfulness affects key physiological measures, including systolic and diastolic blood pressure, and seeks to identify the biomarkers that demonstrate the effectiveness of these practices in managing hypertension. We also explore whether mindfulness and guided meditation can provide lasting benefits for individuals at high risk of cardiovascular diseases, given the emerging evidence these modalities have shown for improving blood pressure regulation.

## Methodology

This study utilized a literature review approach to determine the impact of guided meditation and mindfulness on blood pressure regulation and cardiac biomarkers. A review of existing literature was carried out to evaluate studies related to mindfulness-based interventions (MBIs) and their impact on hypertension and cardiovascular health. PubMed, Cochrane Library, and Google Scholar databases were utilized for the literature review search. The literature study search focused on “mindfulness-based interventions”, “guided meditation”, “hypertension”, “blood pressure”, “cardiac biomarkers”, “cortisol”, “inflammation”, and “heart rate variability”. Academic articles published from 2020 to 2024 were used to ensure relevancy. Additional requirements for the articles included were that they had to be written in English and full-text articles. Given the scarcity of primary RCTs on mindfulness and hypertension within our timeframe, we included meta-analyses to contextualize trends, though we emphasize qualitative synthesis over quantitative pooling.

The study involved extracting key data from each selected study, including study design, sample size, duration of intervention, type of mindfulness-based intervention, and primary outcomes. Data was synthesized to identify patterns, themes, and trends related to the impact of guided meditations and mindfulness on hypertension and cardiac biomarkers.

## An overview of hypertension and its risk factors

Hypertension is a condition in which blood vessel pressure is persistently elevated (≥130/80 mmHg), which can be fatal if not addressed (World Health Organization, 2023). Genetics, older age, overweight or obesity, physical inactivity, drinking too much alcohol and eating a high-salt diet are all risk factors. Hypertension can cause serious complications such as myocardial infarction, stroke, kidney failure, and death (Verma et al., 2021). It is a primary cause of cardiovascular and cerebrovascular mortality. A modest 5 mmHg reduction in SBP has been linked to 14% fewer deaths from stroke, 9% fewer from cardiovascular disease (CVD), and 7% fewer overall mortality. Hypertension guidelines do not recommend initial pharmaceutical treatment for patients with elevated BP and stage 1 hypertension. They focus on lifestyle modifications which include sodium restriction, increased potassium intake, weight loss if overweight/obese, appropriate physical activity, reduction of alcohol intake and a diet rich in fruits, vegetables, whole grains and low-fat dairy products. Pharmaceutical management is reserved for patients with stage 1 hypertension and a history of CVD or a 10-year atherosclerotic cardiovascular disease (ASCVD) risk of 10% or above. For stage 2 hypertension, antihypertensive medication is recommended regardless of ASCVD risk (Flack & Adekola, 2020).

## The physiology of stress and cardiovascular risk

Stress is a common occurrence in human life. Chronic stress has significant clinical implications, including CVD. Stress also raises the incidence and severity of various CVD risk factors, such as, diabetes, hypertension, and obesity (Osborne et al., 2020). For example, the hypothalamic-pituitary-adrenal (HPA) axis, which involves the pituitary and adrenal glands, is activated by stress-induced hypothalamic activity. The anterior pituitary releases ACTH in response to the hypothalamus’ synthesis of vasopressin and corticotropin-releasing factor (CRF), which triggers the adrenal cortex to release glucocorticoids such as cortisol. These glucocorticoids counter-regulate HPA activation and contribute to hypertension, increased adiposity, and insulin resistance (Osborne et al., 2020). On the other hand, stress-induced changes in the sympathetic nervous system (SNS) and parasympathetic nervous system tone cause physiological effects, such as higher blood pressure, vasoconstriction, lower heart rate variability and increased peripheral vascular resistance. According to Mir et al., meditation techniques cause the parasympathetic nervous system to become active, which facilitates relaxation and lessens the negative consequences of stress (Mir et al., 2024).

Additionally, stress can cause a persistent and systemic state of low-grade inflammation, as seen by elevated levels of circulating inflammatory biomarkers such as interferon-gamma (INF-γ), C-reactive protein (CRP) and interleukin-6 (IL-6) (Moreno, 2024). According to a recent meta-analysis by Shah et al., on the impact of yoga and meditation on inflammatory biomarkers in COVID-19 patients, meditation may help patients lower their levels of TNF-α, cortisol, and IL-6 at 2625, 3900, and 1500-minute interventions, respectively, over 8–12 weeks (Shah et al., 2022).

## The role of mindfulness and guided meditation as interventions

Mindfulness is a well-known psychological technique developed by Jon Kabat-Zinn in the 1970s for people experiencing chronic pain. Mindfulness entails cultivating heightened awareness and focused attention on the current moment, particularly the ongoing moment, without passing judgment (Gu et al., 2023). The mindfulness-based stress reduction program operates through eight intensive, week-long sessions to reduce stress, primarily in clinically or non-clinically stressed groups (Mir et al., 2024). The program’s key components emphasize mindfulness during daily tasks such as imaging the body while performing an exercise, conditioning the brain in a specific way while working with (or against) gravity, and moving the body in optimal physical activity choices for meditation and rehabilitation.

Babak et al. conducted a randomized clinical trial study in 2019 in western Iran to evaluate the impact of mindfulness-based stress reduction (MBSR) on mental health, blood pressure, and quality of life (QoL) in adult women with hypertension. Eighty women with Stage I or II hypertension were randomly assigned to two groups: 12 weeks of MBSR and routine care. Blood pressure, stress, anxiety, quality of life and depression were measured using the 36-Item Short Form Survey (SF-36) and Depression, Anxiety, and Stress Scale-21 (DASS-21) questionnaires. According to the findings, the intervention group’s mean systolic and diastolic blood pressures were significantly lower than those of the baseline and control groups. Additionally, the intervention group showed a substantial increase in quality of life, anxiety, stress, and depression scores. The 12-week MBSR program resulted in a significant reduction in blood pressure and improved mental health (Babak et al., 2022).

Further, Mir et al. conducted a systematic review of RCTs on the impact of mindfulness-based meditation on blood pressure in adults with high blood pressure. Nine studies with 543 participants found MBM effective in lowering diastolic and systolic blood pressure. However, there was a gender disparity in seven studies, and four trials did not report the ethnicity of participants. Although the trials’ methodological quality was satisfactory, most of the studies had issues that could compromise the validity of the trials, such as blinding of outcome assessors, absence of allocation concealment, insufficient data reporting, and a high attrition rate. The researchers concluded that MBM therapies could be used as a supportive and early preventive measure for persons with hypertension or increased blood pressure (Mir et al., 2024).

Mindfulness-based interventions (MBIs) have been linked to adaptive increases in heart rate variability (HRV), which is influenced by parasympathetic and sympathetic influences. Vagally-mediated HRV indices, such as time-domain measures, high-frequency HRV, and respiratory sinus arrhythmia, are of particular clinical interest as they indicate the nervous system’s sensitivity to changing physical and emotional demands. Brown et al. meta-analyzed 19 RCTs and found that MBIs were ineffective in increasing HRV compared to control conditions (Brown et al., 2021).

Zhang et al. reviewed 481 studies on cardiovascular risk variables associated with mindfulness-based therapies. The study revealed that MBIs greatly reduced depression and stress and could potentially lessen specific risk factors, thereby averting cardiovascular disease. According to the review, MBIs significantly reduced smoking, glycosylated hemoglobin, binge eating, depression, stress, and both diastolic and systolic blood pressure (Zhang et al., 2023). Table 1 further captures the impact of mindfulness-based interventions on blood pressure, cardiac biomarkers, mental health and quality of life.

**Table 1.** Impact of mindfulness-based interventions on blood pressure, cardiac biomarkers and mental health

Reduction in Blood Pressure	Improvements in Cardiac Biomarkers	Mental Health and Quality of Life
Findings revealed consistent evidence that MBIs significantly reduce blood pressure levels: Babak et al., demonstrated that a 12-week MBSR program significantly reduced SBP and DBP in hypertensive women compared to the control group (Babak et al., 2022). Mir et al. found MBIs to reduce SBP in 8 out of 9 trials effectively and DBP in 6 trials (Mir et al., 2024).	Cortisol Levels: Multiple studies found that mindfulness techniques lowered cortisol levels. Shah et al. found significant reductions in cortisol and pro-inflammatory cytokines (e.g., IL-6) following an 8–12-week intervention (Shah et al., 2022). HRV: Brown et al. observed that, while MBIs may have potential benefits, further research is needed to understand their effect on HRV completely (Brown et al., 2021). Inflammatory markers: Moreno (2024) reported decreased levels of IL-6 and CRP in people who practiced mindfulness, indicating lower systemic inflammation (Moreno, 2024).	MBSR and other interventions improved hypertension patients’ mental health and quality of life. Babak et al. observed substantial reductions in stress, anxiety, and depression levels, which could indirectly contribute to better blood pressure regulation (Babak et al., 2022).

Abbreviations: CRP (C-reactive protein), DBP (diastolic blood pressure), HRV (heart rate variability), IL (interleukin), MBI (mindfulness-based intervention), MBSR (mindfulness-based stress reduction), SBP (systolic blood pressure)

## Discussion

This study aimed to explore the effects of guided meditations and mindfulness interventions on blood pressure regulation and their potential to enhance cardiac biomarkers, including heart rate variability, cortisol levels, and inflammatory markers. The findings tentatively support our thesis that mindfulness and guided meditation could improve blood pressure regulation and cardiac biomarkers, providing a non-pharmacological complement to traditional hypertension and cardiovascular disease treatments.

According to the literature, mindfulness reduces both systolic and diastolic blood pressure. Osborne et al. note that stress-induced hypothalamic-pituitary-adrenal axis and sympathetic nervous system activation in hypertension leads to vasoconstriction, increased cardiac output, and chronic inflammation (Osborne et al., 2020). Meditation and mindfulness engage the parasympathetic nervous system (PNS), which relaxes and regulates the autonomic system, lowering stress and improving emotional regulation (Addington et al., 2020). Like previous studies, Babak et al. discovered that mindfulness meditation alters brain structure, reduces stress, lowers blood pressure, and improves mental health (Babak et al., 2022). Therefore, these strategies can be used to treat clinical diseases and mental health issues, enhancing overall well-being.

MBIs improve cortisol, inflammatory markers, and HRV, biological markers of cardiovascular health. Recognizing these CVD indicators reduces socioeconomic hardship. The interplay of physiological and behavioral risk factors links psychological stress to CVD (Munir & du Toit, 2024). Chronic stress releases glucocorticoids, which increase inflammation and atherosclerotic plaque building. Increased cardiokine secretion induces endothelial dysfunction and atherosclerotic plaques. According to Shah et al., mindfulness has a demonstrated effect on systemic inflammation, as evidenced by significant decreases in cortisol and pro-inflammatory cytokines (Shah et al., 2022). These findings are consistent with the larger studies linking chronic stress and inflammation to increased cardiovascular risk.

Further, heart rate variability (HRV), which evaluates autonomic nervous system adaptation, produced mixed results. While some studies reported no significant benefits, methodological variables may explain variations. Brown et al. conclude that there is currently insufficient evidence to show that MBIs improve vagally mediated HRV compared to control settings (Brown et al., 2021). Future large, well-designed RCTs with low methodological bias could contribute to the current evidence by elucidating whatever function MBIs may play in HRV. The conclusion corresponds with Christodoulou et al.‘s scoping analysis of 17 original MBI research, which found increased HRV reactivity following MBI deployment in non-clinical samples (Christodoulou et al., 2020). The review findings backup HRV as an objective biomarker for proving the effects of MBI; however, further research is needed to address the substantial variability in methodologies among studies.

Analysis revealed that mindfulness improves mental health by lowering stress, anxiety, and depression, which indirectly supports better cardiovascular health. (Mir et al., 2024; Verma et al., 2021; Osborne et al., 2020). Psychological stress is a known cause of hypertension; thus, MBIs have an important role in improving cardiac biomarkers and lowering cardiovascular risk. The strong quality-of-life gains demonstrated by Babak et al. emphasize the benefits of MBIs in hypertension management.

The study’s findings indicate that guided meditation and mindfulness can complement traditional hypertension therapies, offering cost-effective and value-for-money benefits (Babak et al., 2022). MBIs can be provided in group format or as self-help interventions and can be integrated into educational programs for clinicians and professionals. While benefits are similar to cognitive behavioral interventions, mindfulness requires less professional training, takes less time for workers and clients, and is less expensive. Lifestyle Medicine is a rapidly evolving field that has been gaining more traction in recent years. It essentially focuses on evidence-based practices to prevent, treat and manage chronic conditions by incorporating a holistic approach to patient care; focusing on nutrition, exercise, sleep quality, substance use reduction, social connections, environmental factors and stress management. Chronic stress can inevitably lead to disease and exacerbate pre-existing conditions, making its reduction more crucial than ever before. As a result, techniques such as mindfulness, meditation, deep breathing exercises, and relaxation therapies are employed to reduce anxiety and stress.

The present study indeed has limitations, including study design variability, sample size, and intervention protocols. Notably, the included studies exhibit methodological limitations that temper the strength of our findings. For instance, small sample sizes (e.g. Babak et al’.s trial with 80 participants) and lack of blinding outcome assessment (Mir et al.) may inflate effect estimates. In addition, given the absence of standardized procedures and settings for MBIs in the included research studies, there was a significant variation in the duration and specific methods of the interventions they were administered. While incorporating lifestyle medicine principles into hypertension management emphasizes the importance of holistic, personalized care that goes beyond pharmacological treatments, it is important to note that mindfulness-based interventions can be significantly influenced by individual receptiveness as well. Patients who believe in the efficacy of meditation may experience stronger physiological benefits, including reduced blood pressure and improved cardiac biomarkers (Buric et al., 2022). This mind-body connection essentially parallels the placebo effect, which has indeed demonstrated measurable outcomes in cardiovascular health. Additionally, variations in response based on gender, race, and age must be explored further to tailor interventions as needed. These factors underscore the need for cautious interpretation and highlight gaps for future RCTs since understanding these variables is critical to advancing personalized medicine and ensuring equitable access to effective, non-pharmacological hypertension treatments.

Furthermore, most studies identified were small trials involving a diverse geographical population with varying hypertension levels and racial backgrounds. Future research should prioritize diverse, representative samples to ensure findings can be generalized across different populations. While the limited number of included studies precludes definitive conclusions, the consistency of blood pressure reductions across studies supports the potential of MBIs as adjunctive therapies. Further long-term studies are indeed necessary to evaluate the sustainability of benefits and potential for cardiovascular event reduction.

## Conclusion

Guided meditations and mindfulness-based treatments show preliminary promise for non-pharmacological hypertension therapy and improving cardiovascular biomarkers, albeit there is no doubt that their efficacy requires validation in larger and more diverse samples. These strategies evidently minimize stress and inflammation and enhance autonomic balance, improving blood pressure regulation and mental wellness. Consistent reductions in SBP and DBP, as well as improvements in cortisol and inflammatory markers, show that mindfulness can indeed address the complicated nature of hypertension. However, further research is required to standardize intervention techniques, investigate long-term impacts, and guarantee that various populations are represented. Integrating these strategies into clinical practice can improve hypertension care and empower patients to participate actively in their own health.

## Data Availability

Not applicable since no new data was generated.

## References

[cit0001] Addington, E. L., Javandel, S., De Gruttola, V., Paul, R., Milanini, B., Ances, B. M., Moskowitz, J. T., & Valcour, V. (2020). Mindfulness-based stress reduction for HIV-associated neurocognitive disorder: Rationale and protocol for a randomized controlled trial in older adults. *Contemporary Clinical Trials*, 98, 106150. 10.1016/j.cct.2020.106150 32942053 PMC7686285

[cit0002] Babak, A., Motamedi, N., Mousavi, S. Z., & Ghasemi Darestani, N. (2022). Effects of mindfulness-based stress reduction on blood pressure, mental health, and quality of life in hypertensive adult women: A randomized clinical trial study. *The Journal of Tehran University Heart Center*, 17(3), 127–133. 10.18502/jthc.v17i3.10845 37252082 PMC10222936

[cit0003] Brown, L., Rando, A. A., Eichel, K., Van Dam, N. T., Celano, C. M., Huffman, J. C., Morris, M. E. (2021). The effects of mindfulness and meditation on vagally mediated heart rate variability: A meta-analysis. *Psychosomatic Medicine*, 83(6), 631–640. 10.1097/PSY.0000000000000900 33395216 PMC8243562

[cit0004] Buric, I., Farias, M., Driessen, J. M. A., Brazil, I. A. (2022). Individual differences in meditation interventions: A meta-analytic study. *British Journal of Health Psychology*, 27(3), 1043–1076. 10.1111/bjhp.12589 35224829 PMC9543193

[cit0005] Christodoulou, G., Salami, N., Black, D. S. (2020). The utility of heart rate variability in mindfulness research. *Mindfulness*, 11(3), 554–570. 10.1007/s12671-019-01296-3

[cit0006] Flack, J. M., Adekola, B. (2020). Blood pressure and the new ACC/AHA hypertension guidelines. *Trends in Cardiovascular Medicine*, 30(3), 160–164. 10.1016/j.tcm.2019.05.003 31521481

[cit0007] Gu, J. J., Tong, X. S., Meng, S. S., Xu, S.-H., & Huang, J.-Y. (2023). Effect of mindfulness-based stress reduction in patients with acute myocardial infarction after successful primary percutaneous coronary intervention: A retrospective study. *BMC Cardiovascular Disorders*, 23(1), 315. 10.1186/s12872-023-03346-0 37353727 PMC10290389

[cit0008] Li, Y., Buys, N., Li, Z., Li, L., Song, Q., & Sun, J. (2021). The efficacy of cognitive behavioral therapy-based interventions on patients with hypertension: A systematic review and meta-analysis. *Preventive Medicine Reports*, 23, 101477. 10.1016/j.pmedr.2021.101477 34285871 PMC8278424

[cit0009] Mills, K. T., Stefanescu, A., He, J. (2020). The global epidemiology of hypertension. *Nature Reviews Nephrology*, 16(4), 223. 10.1038/s41581-019-0244-2 32024986 PMC7998524

[cit0010] Mir, I. A., John, A. T., Humayra, S., Khan, Q. I., Chong, T. F., & Manan, H. A. (2024). Effect of mindfulness-based meditation on blood pressure among adults with elevated blood pressure and hypertension: A systematic review of randomized controlled trials. *Complementary Therapies in Medicine*, 85, 103084. 10.1016/j.ctim.2024.103084 39277117

[cit0011] Moreno, J. J. (2024). Modulation of inflammatory response and pain by mind-body therapies such as meditation. *Brain, behavior, and Immunity - Integrative*, 5, 100036. 10.1016/j.bbii.2023.100036

[cit0012] Munir, L. Z., du Toit, E. F. (2024). Impact of chronic psychological stress on cardiovascular disease risk: A narrative review. *Heart and Mind*, 8(4), 268–278. 10.4103/hm.HM-D-24-00040

[cit0013] Osborne, M. T., Shin, L. M., Mehta, N. N., Pitman, R. K., Fayad, Z. A., & Tawakol, A. (2020). Disentangling the links between psychosocial stress and cardiovascular disease. *Circulation: Cardiovascular Imaging*, 13(8). 10.1161/CIRCIMAGING.120.010931 PMC7430065 32791843

[cit0014] Shah, K., Adhikari, C., Sharma, S., Saha, S., Saxena, D., & Garrido, G. (2022). Yoga, meditation, breathing exercises, and inflammatory biomarkers with possible implications in COVID-19: A systematic review and meta-analysis of randomized controlled trials. *Evidence-Based Complementary and Alternative Medicine*, 2022, 1–28. Article 3523432. 10.1155/2022/3523432

[cit0015] Verma, N., Rastogi, S., Chia, Y. C., Siddique, S., Turana, Y., Cheng, H. M., Sogunuru, G. P., Tay, J. C., Teo, B. W., Wang, T.-D., Tsoi, K. K. F., & Kario, K. (2021). Non-pharmacological management of hypertension. *Journal of Clinical Hypertension*, 23(7), 1275–1283. 10.1111/jch.14236 33738923 PMC8678745

[cit0016] World Health Organization. (2021). Guideline for the pharmacological treatment of hypertension in adults [Internet]. World Health Organization. Recommendations. https://www.ncbi.nlm.nih.gov/books/NBK573627/34495610

[cit0017] World Health Organization. (2023, March 16). Hypertension. https://www.who.int/news-room/fact-sheets/detail/hypertension

[cit0018] Zhang, X. F., Li, R. N., Deng, J. L., Chen, X. L., Zhou, Q. L., Qi, Y., Zhang, Y.-P., & Fan, J. M. (2023). Effects of mindfulness-based interventions on cardiovascular risk factors: An umbrella review of systematic reviews and meta-analyses. *Journal of Psychosomatic Research*, 177, 111586. 10.1016/j.jpsychores.2023.111586 38185037

